# COVID-19 in Kidney Transplant Recipients With Diabetes Mellitus: A Propensity Score Matching Analysis

**DOI:** 10.3389/ti.2022.10375

**Published:** 2022-07-25

**Authors:** Érika B. Rangel, Débora D. de Lucena, Isabella Aguiar-Brito, Luís Gustavo Modelli de Andrade, Alexandre Veronese-Araújo, Marina P. Cristelli, Hélio Tedesco-Silva, José O. Medina-Pestana

**Affiliations:** ^1^ Hospital do Rim, São Paulo, Brazil; ^2^ Nephrology Division, Federal University of São Paulo, São Paulo, Brazil; ^3^ Hospital Israelita Albert Einstein, São Paulo, Brazil; ^4^ Department of Internal Medicine, Botucatu Medical School, University of São Paulo State, Botucatu, Brazil

**Keywords:** COVID-19, diabetes mellitus, outcomes, kidney transplant, propensity score

## Abstract

Kidney transplant recipients present higher rates of pre-existing comorbidities, in particular diabetes mellitus (DM), hypertension, and cardiac disease. We aimed to verify the main risk factors related to DM that contribute to COVID-19 progression and mortality in a kidney transplant setting. From March to August 2020, we evaluated 300 kidney transplant recipients affected by COVID-19. We used propensity score matching (PSM) to estimate the impact of DM on COVID-19. After matching, all baseline characteristics were well balanced between those with and without DM (*n* = 100 in each group). Case fatality rate, the requirement of invasive mechanical ventilation (IMV), and acute kidney injury (AKI) were associated with previous fasting blood glucose, and C-reactive protein (CRP), and lactate dehydrogenase (LDH) levels on admission. These findings were similar in kidney transplant patients with and without DM. Glycemia on admission and estimated glomerular filtration rate (eGFR) either on admission or basal correlated to the need of IMV and development of AKI, respectively. Poor glycaemic control, eGFR, markers of inflammation (CRP) and tissue damage (LDH) were indicative of COVID-19 burden in kidney transplant recipients and may be useful tools for risk-stratifying this population, independently of the DM status, during the pandemic.

## Graphical Abstract

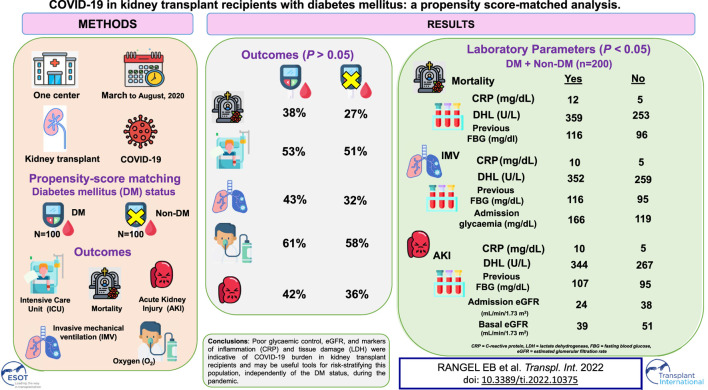

## Introduction

The cardio-metabolic disease is associated with increased mortality and severity of coronavirus disease 2019 (COVID-19) pneumonia, including the transfer to intensive care unit (ICU), invasive mechanical ventilation (IMV), acute kidney injury (AKI), and death [1–4]. Cardio-metabolic disease encompasses broad pathological changes, such as insulin resistance, diabetes mellitus (DM), dyslipidemia, abdominal obesity, and hypertension, and environmental risk factors such as smoking, sedentary lifestyle, poor diet, and poverty. The ultimate consequences of that combination are higher rates of viral entrance, direct viral toxicity, endothelial dysfunction, thrombi-inflammation, dysregulation of the immune response, and derangement of the renin-angiotensin-aldosterone system [5].

The data describing the outcomes of solid-organ transplantation (SOT) recipients with COVID-19 has raised a debate in the literature on whether transplantation *per se* was a major risk for COVID-19 progression and mortality, or whether the presence of cardiometabolic comorbidities was the main factor responsible for the adverse outcomes [6]. Therefore, the initial reports highlighted high rates of AKI (37.8%–52.1%), transfer to ICU (33.8%–36%), respiratory failure requiring intubation (27%–29.6%), and case fatality rate (CFR; 18.7%–32%) in these population [7–9]. Importantly, a high prevalence of pre-existent comorbidities was equally documented, such as hypertension (77.4%–95.1%), DM (41.3%–52.1%), obesity/overweight (35.1%–63.8%), heart disease (21.8%–36.2%) and lung disease (10.4%–18.8%), as well as age >60–65 years-old (29.3–56.2%) and male gender (61.2–66%) in SOT setting [7–9]. When compared to non-SOT individuals, SOT individuals had increased odds of receiving IMV (2.34), developing AKI (2.41), being transferred to ICU (1.46), and mortality (1.94) [10].

Despite the growing literature focusing on the prognosis of COVID-19 in transplant recipients, data on selected high-risk clinical populations that merit special consideration, such as immunocompromised individuals with a history of DM, remain undetermined. Diabetic individuals are susceptible to a substantial burden of micro and macrovascular complications [11] and dysregulation of the immune system [12], which could predispose them to an increase in COVID-19 severity and mortality. Here, we set out to verify the clinical manifestations, outcomes, and CFR in a population of kidney transplant recipients with DM and the diagnosis of COVID-19 using the propensity-score matched analyses in a single center.

## Patients and Methods

### Study Design and Setting

A cohort, cross-sectional, observational, and descriptive study was conducted at Hospital do Rim, São Paulo, SP, Brazil. The medical records of patients who were either hospitalized or non-hospitalized with the diagnosis of COVID‐19 during the study period of March to August 2020 were assessed, corresponding to the first wave of COVID-19 in Brazil. We included only patients in whom SARS‐CoV‐2 was detected by nasopharyngeal swab RT‐PCR (reverse transcriptase-polymerase chain reaction). The population at risk included 11,875 kidney transplant patients undergoing outpatient follow-up [13]. Of 590 kidney transplant recipients who became ill, 300 were included in the study. Six were excluded for being a double transplant, 4 for having lost the graft in the period before COVID-19, 4 for being a recent transplant and being in delayed graft function at the time of diagnosis of COVID-19, 1 for not using immunosuppressive drugs due to cancer treatment, 1 for being underage and 274 were excluded for missing data due to admission to other services (Supplementary Figure S1).

A standardized data collection form was developed to retrospectively retrieve relevant information from medical records. Data were collected regarding patient demographics and laboratory parameters on admission with COVID-19 symptoms. The last patient was included in the study on 30th August 2020. The Ethics and Research Committee of the Federal University of São Paulo (CAEE 35311020.9.0000.8098) approved the study. Informed consent was obtained from all patients, whereas a waiver was granted for patients who died in other hospitals.

Patient demographics include age, sex, race, body mass index (BMI), type of donor, time of transplant, as well as the presence of comorbidities (smoking, hypertension, DM, chronic obstructive pulmonary disease [COPD], heart disease, liver disease, and autoimmune disease) were collected. We also evaluated the symptoms on admission.

Diabetes was defined according to the use of insulin and/or oral antidiabetics, hypertension and whether individuals were on anti-hypertensive drugs, liver disease and whether hepatitis B or C were diagnosed, and heart disease and whether heart failure and/or coronary artery disease were present.

### Laboratory Testing

On admission, we evaluated in-hospital laboratory data: lymphocytes, serum creatinine, glycemia, aspartate aminotransferase (AST), alanine aminotransferase (ALT), D-dimer, lactate dehydrogenase (LDH), and C-reactive protein (CRP). As for laboratory data before admission, we collected baseline creatinine (mean the last three measurements), fasting blood glucose (FBG; last measurement within 6 months), and glycated hemoglobin (HbA_1c_; last measurement within the 1 year).

The estimated glomerular filtration rate (eGFR) was calculated using the formula defined in the CKD-EPI (Chronic Kidney Disease Epidemiology Collaboration) study: 175 × serum creatinine^−1.154^ × age^− 0.203^ × 1.212 [if black] × 0.742 [if woman], where the glomerular filtration rate or GFR is expressed in ml/min/1.73 m^2^ of the body surface [14].

### Statistical Analysis

Two groups of renal receptors affected by COVID-19, e.g., diabetic or DM (+) and non-diabetic or DM (−), were separated and the outcomes were then evaluated, based on death, transfer to ICU, AKI classified in accordance do KDIGO guidelines [15], need for hemodialysis (HD) and supplemental oxygen (O_2_), and IMV.

Independent samples *t-*test and Chi‐square test were used to identify the association between DM and demographic and laboratory parameters, and the outcomes previously mentioned. Data were described as mean ± standard deviation or median and interquartile range, as indicated. Frequencies and percentages were reported for qualitative data.

**FIGURE 1:**
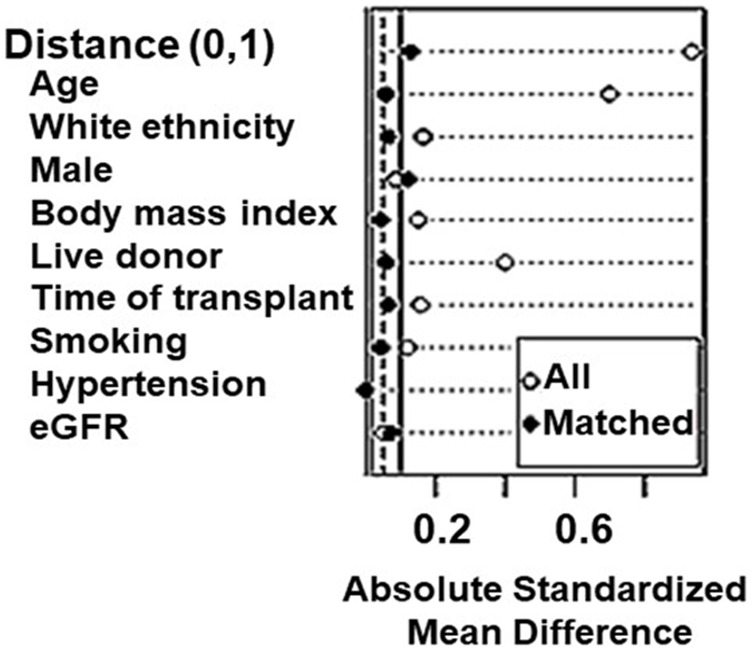
Assessment of baseline characteristics for propensity-score matching (PSM) analyses in kidney transplant recipients.

Next, we used propensity score matching (PSM) to estimate the effect of the group accounting for confounding by the included covariates. We included in match the variables associated with COVID-19 prognosis by previous reports: age, sex, race, BMI, hypertension, time after transplantation, smoking, and eGFR. We used 1:1 nearest neighbor PSM without replacement with a caliper of 0.2, which yielded adequate balance (Figure 1). The propensity score was estimated using a logistic regression of the treatment (non-diabetes/diabetes) on the covariates. After matching, all standardized mean differences for the covariates were below 0.1 indicating adequate balance.

We performed a Cox regression before matching to evaluate the association between DM and covariates with 60-days death. Importantly, we included the same covariates used for PSM analysis. We did not perform Cox regression with the outcomes of ICU admission and hospitalization because these could introduce an immortal time bias. For matched cohort, we performed a Kaplan-Meier analysis of 60-days death.

Data were analyzed using IBM^®^ SPSS (Statistical Product and Services Solutions, version 18.0, SPSS Inc., Chicago, IL, United States). A *P*-value of <0.05 was considered significant for all data analyses.

## Results

In our kidney transplant population of 300 patients, 57.3% were men (*n* = 172), the mean age was 52.5 ± 12.2 years, 71.6% (*n* = 215) were deceased-donor kidney transplant recipients, mean time of transplant was 94.1 ± 71.6 months (Supplementary Table S1). A total of 228 (76%) patients required hospitalization and the average length of stay was 23 ± 22 (median 15 days) and 89 (29.6%) deaths were registered. Immunosuppressive regimen was mainly based on Tacrolimus (TAC) and Mycophenolate (MPA) (*n* = 152; 50.6%), TAC and Azathioprine (AZA) (*n* = 49; 16.3%), TAC and mTOR inhibitor (mTORi) (*n* = 24; 8%), AZA and Cyclosporine A (CSA) (*n* = 22; 7.3%). All patients were using steroids as part of their immunosuppressive regimen.

Among the individuals included in the study, 117 (39%) were diabetic, and these individuals were older (56.9 ± 10.3 *versus* 49.6 ± 12.5 years old), had more hypertension (85.5% *versus* 67.8%), and heart disease (17.9% *versus* 6%), and had more often received a kidney from deceased donors (81.2% *versus* 65.6%) (all *p* < 0.05; Supplementary Table S1). From a clinical perspective, we observed that anosmia was found more frequently in non-diabetics on admission (34.4% *versus* 22.2%, *p* = 0.025) (Supplementary Table S1).

Analyses of laboratory data disclosed poor glycaemic control and higher levels of CRP in diabetic individuals. Conversely, no differences in eGFR, LDH, lymphocytes, D-dimer, or liver tests were found between diabetic and non-diabetic kidney transplant recipients (Table 2S). Among the 300 kidney transplant recipients, 46.7% required ICU admission, 54.3% used supplemental O_2_, 34% needed IMV, 58% developed AKI, 36.3% underwent HD, and 29.7% died (Supplementary Table S2). When analyzing the subgroup of diabetic kidney transplant recipients (*n* = 117), we found that the CFR was 39.3% and higher rates of COVID-19 progression were noticed, including ICU admission (54.7%), the requirement of supplemental O_2_ (61.5%), and IMV (44.4%), development of AKI stage 3 (47%) and the need for HD (43.6%) (all *p* < 0.05; Supplementary Table S2).

Next, we applied the PSM and paired 1:1 (diabetic and non-diabetic) and balanced all baseline characteristics (Table 1). After matching, we obtained a total of 200 patients (*n* = 100 diabetics and *n* = 100 non-diabetics). In this matched population, CFR, the requirement for IMV or O_2_, development of AKI, and the need for HD were similar between diabetic and non-diabetic kidney transplant recipients (Table 1). Overall, CFR was 32.5%.

**TABLE 1:** Demographic variables and outcomes after applying the propensity-score matching (PSM) for kidney transplant recipients with diabetes mellitus (DM) and without DM.

Variables and outcomes	DM (−) (N = 100)	DM (+) (N = 100)	*P*
Age (median, IQR)	54 (47, 63)	56 (50, 62)	0.5
White ethnicity (n, %)	60 (60)	57 (57)	0.7
Male (n, %)	59 (59)	53 (53)	0.4
BMI (median, IQR)	27.3 (23.8, 29.7)	28.0 (24.3, 30.7)	0.6
Living donor (n,%)	23 (23%)	21 (21%)	0.7
Transplant time (months) (median, IQR)	64 (30, 143)	70 (36, 122)	>0.9
Smoking (n, %)	22 (27%)	24 (29%)	0.8
Hypertension (n, %)	83 (83%)	83 (83%)	>0.9
Basal eGFR (median, IQR)	47 (30, 60)	48 (31, 65)	0.8
Death (n, %)	27 (27%)	38 (38%)	0.10
IMV (n, %)	32 (32%)	43 (43%)	0.11
HD (n, %)	36 (36%)	42 (42%)	0.4
ICU (n, %)	51 (51%)	53 (53%)	0.8
O_2_ (n, %)	58 (58%)	61 (61%)	0.7

IQR, interquartile range; BMI, body mass index; eGFR, estimated filtration glomerular rate in mL/min/1.73 m^2^; IMV, invasive mechanical ventilation; HD, hemodialysis; ICU, intensive care unit; O_2_, oxygen. After applying the PSM, 83 non-diabetic patients and 17 diabetic patients were excluded.

Evaluation of the laboratory data indicated that FBG previous to admission, CRP, and LDH levels on admission were related to an increased risk of death, the requirement of IMV, and the development of AKI in the kidney transplanted population (Table 2).

**TABLE 2:** Outcomes in kidney transplant recipients with diabetes mellitus (DM) and without DM after applying the propensity-score matching (PSM).

Laboratory data	Alive (N = 135)	Not Alive (N = 65)	*P*
Previous FBG (mg/dl)	96 (86, 121)	116 (93, 194)	***<0.001***
Glycemia on admission (mg/dl)	124 (95, 217)	156 (112, 252)	0.086
Previous Hb1Ac (%)	6.20 (5.50, 7.80)	6.80 (5.60, 8.60)	0.2
CRP (mg/dl)	5 (2, 11)	12 (5, 18)	***<0.001***
LDH (U/L)	253 (217, 344)	359 (288, 483)	***<0.001***
eGFR on admission	34 (22, 50)	31 (17, 46)	0.3
Basal eGFR	47 (32, 63)	49 (27, 59)	0.5
	**IMV (−) (N = 125)**	**IMV (+) (N = 75)**	
Previous FBG (mg/dl)	95 (84, 114)	116 (93, 190)	***<0.001***
Glycemia on admission (mg/dl)	119 (95, 181)	166 (115, 272)	***0.009***
Previous Hb1Ac (%)	6.20 (5.50, 7.55)	6.80 (5.60, 8.70)	0.10
CRP (mg/dl)	5 (2, 11)	10 (4, 16)	***0.003***
LDH (U/L)	259 (220, 337)	352 (257, 485)	***<0.001***
eGFR on admission	34 (21, 50)	31 (18, 46)	0.2
Basal eGFR	47 (31, 64)	47 (28, 59)	0.5
	**AKI (−) (N = 122)**	**AKI (+) (N = 78)**	
Previous FBG (mg/dl)	95 (85, 135)	107 (92, 166)	***0.004***
Glycemia on admission (mg/dl)	137 (95, 215)	150 (108, 256)	0.2
Previous Hb1Ac (%)	6.20 (5.55, 7.85)	6.60 (5.50, 8.60)	0.5
CRP (mg/dl)	5 (2, 11)	10 (3, 15)	***0.023***
LDH (U/L)	267 (223, 342)	344 (236, 438)	***0.005***
eGFR on admission	38 (26, 52)	24 (13, 43)	***<0.001***
Basal eGFR	51 (35, 67)	39 (22, 56)	***<0.001***

All values are median and interquartile range. FBG, fasting blood glucose; Hb1Ac, glycated hemoglobin; CRP, C-reactive protein; LDH, lactate dehydrogenase; eGFR (in mL/min/1.73 m^2^), estimated glomerular filtration rate; IMV, invasive mechanical ventilation; AKI, acute kidney injury. The bold-italic values mean that they are statiscally significant (*p* < 0.05).

In addition to the variables aforementioned, glycemia on admission was associated with the requirement of IMV, which was observed in 37.5% of the kidney transplanted patients (Table 2). Likewise, basal and admission eGFR was associated with AKI development in 39% of both diabetic and non-diabetic patients (Table 2).

In transplanted patients with DM (N = 100), 38% died. Previous FBG to admission and LDH on admission were associated with CFR (Table 3). In addition, 43% of diabetic patients required IMV. Not only previous FBG to admission but also higher levels of glycemia and LDH levels on admission were associated with the need for IMV (Table 3).

**TABLE 3:** Outcomes in kidney transplant recipients with diabetes mellitus (DM) after applying the propensity-score matching (PSM).

Laboratory data	ALIVE (N = 62)	Not ALIVE (N = 38)	P
Previous FBG (mg/dl)	114 (90, 167)	169 (119, 249)	***<0.001***
Glycemia on admission (mg/dl)	186 (109, 248)	224 (186, 327)	0.14
Previous Hb1Ac (%)	7.45 (6.20, 9.40)	8.20 (6.80, 9.40)	0.2
CRP (mg/dl)	7 (2, 13)	11 (5, 20)	0.062
LDH (U/L)	250 (214, 352)	352 (292, 492)	***0.001***
eGFR on admission	34 (21, 48)	34 (19, 46)	0.7
Basal eGFR	46 (32, 62)	51 (25, 69)	0.8
	**IMV (−) (N = 57)**	**IMV (+) (N = 43)**	
Previous FBG (mg/dl)	113 (90, 166)	168 (119, 247)	***<0.001***
Glycemia on admission (mg/dl)	164 (100, 238)	236 (190, 333)	***0.017***
Previous Hb1Ac (%)	7.40 (6.20, 9.30)	8.35 (6.80, 9.78)	0.10
CRP (mg/dl)	7 (2, 13)	10 (3, 18)	0.15
LDH (U/L)	265 (211, 350)	344 (256, 490)	***0.005***
eGFR on admission	34 (20, 49)	34 (20, 46)	0.6
Basal eGFR	47 (32, 64)	51 (27, 64)	>0.9
	**AKI (−) (N = 58)**	**AKI (+) (N = 42)**	
Previous FBG (mg/dl)	120 (91, 169)	160 (114, 249)	***0.008***
Glycemia on admission (mg/dl)	204 (148, 244)	224 (140, 333)	0.3
Previous Hb1Ac (%)	7.50 (6.30, 9.20)	8.05 (6.65, 9.85)	0.4
CRP (mg/dl)	8 (2, 13)	9 (3, 18)	0.4
LDH (U/L)	279 (222, 354)	340 (232, 427)	0.093
eGFR on admission	36 (27, 50)	28 (12, 46)	***0.017***
Basal eGFR	50 (34, 68)	47 (22, 56)	0.092

All values are median and interquartile range. Hb1Ac, glycated hemoglobin; CRP, C-reactive protein; LDH, lactate dehydrogenase; eGFR (in mL/min/1.73 m^2^), estimated glomerular filtration rate; IMV, invasive mechanical ventilation; AKI, acute kidney injury. The bold-italic values mean that they are statiscally significant (*p* < 0.05).

When evaluating AKI outcomes in kidney transplant recipients with DM, we found that 42% of these individuals developed any stage of kidney dysfunction. Previous FBG to admission and eGFR on admission were related to AKI occurrence (Table 3).

To note, Cox regression analysis performed pre-PSM showed no association between DM and 60-days death (Supplementary Table S3), indicating similar results to those observed post-PSM. The analysis was performed as recommended after matching using weights.

**FIGURE 2:**
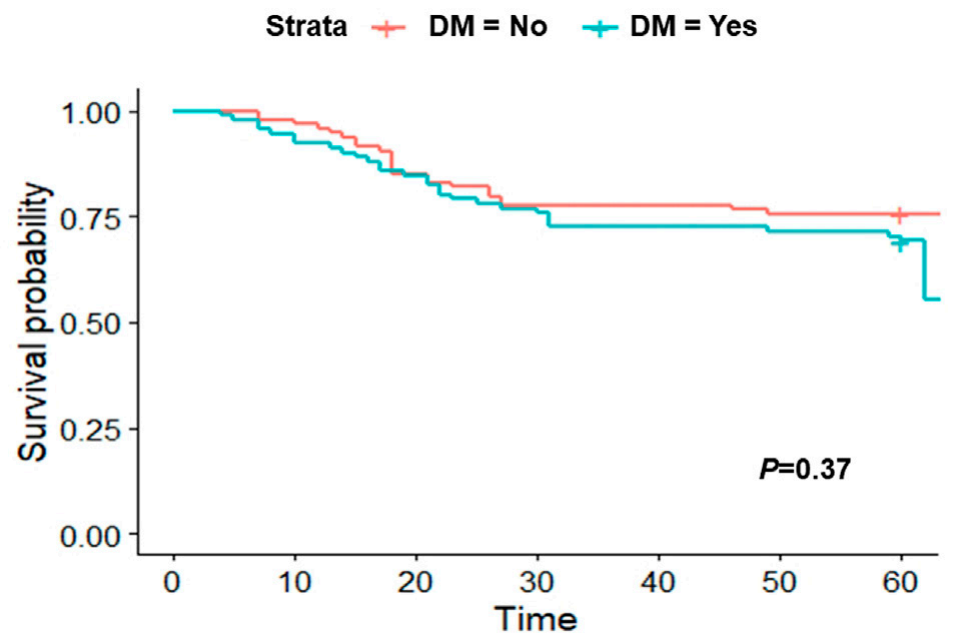
Kaplan-Meier curve after applying the propensity-score matching (PSM) at 60 days.

For matched cohort, a Kaplan-Meier analysis showed no association between DM and 60-days death (*p* = 0.37; Figure 2).

Importantly, the burden of immunosuppression was not different between DM and non-DM patients after PSM, including trough levels and doses (Supplementary Table S4). In both groups, no patients used thymoglobulin or steroid pulse in the past 3 months. In fact, the majority of the patients were in a stable maintenance phase. Likewise, modification of the immunosuppressive regimen was not different between groups and was performed in almost two-thirds of the patients (Supplementary Table S4).

## Discussion

Here, to gain insight into the impact on outcomes when potentially severe conditions are combined, we have outlined the analysis of the subgroup of kidney transplant recipients with cardio-metabolic disease, in particular DM, and the potential of this combination to worsen COVID-19 progression and increase CFR. We found an overall CFR of 32.5%, which is in accordance with previous studies with immunocompromised individuals in transplant settings (27%–32%) [7,8,16]. The diabetic population, in particular, presented an increased CFR (38%), although not significantly different from kidney transplant recipients without DM after applying the PSM.

In our study, kidney transplant recipients with DM were older, and exhibited higher rates of hypertension and heart disease, which put them at higher risk of COVID-19 progression and mortality, as described in non-transplanted individuals with long-term DM and newly diagnosed DM [17–19].

After matching, we observed that for both groups of kidney transplant recipients, diabetic and non-diabetic, previous glycemic control and glycemia on admission, the inflammatory marker CRP and tissue damage marker LDH, as well as function were indicative of severity. Therefore, markers of coagulation and liver tests were not useful tools to stratify the risk of kidney transplant recipients diagnosed with COVID-19, in contrast to non-transplanted individuals [20].

Age, a non-modifiable variable, is associated with increased mortality from COVID-19 in the general population [21] and transplanted populations [6–8,16], as we also observed in our study. Likewise, age is related to COVID-19 progression, in particular AKI development [6,7,16]. In the general population, AKI developed in one-third of hospitalized patients with COVID-19 and the independent risk factors for its development included advanced age, black race, hypertension, DM, cardiovascular disease, use of vasopressor, and need for ventilation [22]. Furthermore, elevated values of creatinine and blood urea nitrogen, any stage of AKI-KDIGO, proteinuria, and haematuria were independent risk factors for in-hospital mortality, even after adjusting for demographic and laboratory variables [23,24].

In our population, AKI occurred in 39% of the kidney transplant recipients after applying the PSM. AKI was reported in 52% of kidney transplant recipients in TANGO International Transplant Consortium, whereas mechanical ventilation was required in 29% [7]. In this study, a high incidence of comorbidities was also present, including hypertension (95%), DM (52%), obesity (49%), and cardiac disease (28%). To note, age was greater than observed in our study. Furthermore, higher rates of mortality were associated not only with age but also with lymphocyte count, GFR, LDH, procalcitonin, and IL-6 levels [7].

In elderly individuals, AKI, IL-6 levels, and myocardial injury were equally associated with mortality, indicating the burden of COVID-19 with aging [25]. AKI occurs not only through direct damage to podocytes and tubular epithelial cells by SARS-CoV-2, but also through the inflammatory milieu, in particular the cytokine storm, and other causes, including rhabdomyolysis, cardio-renal syndrome, and secondary infections [26]. Post-mortem kidney analyses disclosed acute tubular injury in almost all cases and less frequently thrombi and collapsing segmental and focal glomerulosclerosis associated with the APOL1 variant [27]*.* These findings were in agreement with the histological features of kidney biopsies performed in COVID-19 patients with AKI or proteinuria and obtained from transplanted and non-transplanted individuals [28]. Importantly, recovery of kidney allograft function due to COVID-19 occurs in only 40% of the kidney transplant recipients and is associated with GFR and proteinuria on admission, previous rejection, higher SOFA score, hypotension, and KDIGO stage 3 [29].

To note, AKI is primarily seen in COVID-19 patients with respiratory failure, with almost 90% of patients on IMV developing AKI of any stage of KDIGO compared to less than 25% of non-ventilated patients, indicating a temporal relation between AKI and respiratory failure [22], as we also observed in our population. Thus, the clinical-laboratory score for risk stratification of patients showed that DM, PaO_2_/FiO_2_ ratio, and the inflammatory and endothelial dysfunction markers CRP and LDH are predictive for IMV requirement [30].

In our study, diabetic individuals were older and had greater cardio-metabolic comorbidity burden, in particular hypertension and cardiac disease, as reported elsewhere [19]. Not only long-term DM and newly diagnosed DM [19] but also hyperglycemia are associated with ICU admission, the need for IMV, and death [31-33]. In diabetic individuals, including newly-diagnosed DM, admittance glucose levels correlated to clinical markers, including respiratory (higher respiratory rate and lower SatO_2_ and PaO_2_/FIO_2_ ratio) and hemodynamic (higher levels of systolic blood pressure) parameters [19,33] and inflammatory (CRP, IL-6, and procalcitonin), hematologic (leucocytosis, lymphopenia, anemia, and thrombocytopenia), and tissue damage (D-dimer, ALT, troponin, and lactate) markers^19^. Admission hyperglycemia may result from an enhanced response of counter-regulatory hormones and cytokine storm exacerbating insulin resistance [34], which adversely impact the immune response. Thus, diabetic individuals present more frequently lymphopenia and higher levels of cytokines IL-2R, IL-6, IL-8, IL-10, CRP, procalcitonin, and TNF-α, as well as the distinctly reduced Th1/Th2 cytokines ratios and reduced peripheral numbers of CD8^+^ T lymphocytes and NK cells when compared to non-diabetic individuals [35,36], which may lead to longer hospitalization time and SARS-CoV-2 shedding [37]. Therefore, exacerbated inflammatory responses within 24 h of admission correlate with COVID-19 severity in diabetic individuals, in particular IL-6 and LDH, whose longitudinal analyses hold an association with worse outcomes [38]. Additionally, FBG ≥126 mg/dl on admission in patients with COVID-19 without a previous diagnosis of DM is associated with an elevated risk of ICU admission, IMV, and death [19,32,39].

In our study, the median values of glycemia on admission in renal transplant recipients, independently of the DM status, were associated with IMV requirement (166 mg/dl *versus* 119 mg/dl). In diabetic patients, higher values of glycemia on admission were equally associated with a worse respiratory outcome (236 mg/dl *versus* 164 mg/dl). Furthermore, higher levels of the previous FBG were associated with COVID-19 severity, including, the development of AKI, the need for IMV, and CFR in both diabetic and non-diabetic kidney transplant recipients. Elevated glucose levels may regulate SARS-COV-2 replication and cytokine production, trigger mitochondrial reactive oxygen species production, and promote glycolysis in monocytes [40]. These cells are the most enriched immune cell types in the lungs of COVID-19 patients and play an important role in the pathogenicity of the disease. Monocyte-derived cytokines drive T lymphocyte dysfunction and, ultimately, may lead to cell death from diverse organs. Importantly, even after glucose control in diabetics, the macrophage is dysfunctional in these patients, exhibiting M1 pro-inflammatory phenotype and elevated levels of inflammatory chemokines CXCL1, CXCL5, and RANTES^12^. Therefore, adequate long-term glycaemic control and early identification of post-transplant DM is of paramount importance to decrease the inflammatory milieu and, ultimately, the severity of COVID-19.

In addition, pre-existing cardio-metabolic comorbidities found in kidney transplant recipients, such as DM, hypertension, and cardiac disease, are associated with chronic endothelial dysfunction. SARS-CoV-2 can directly infect endothelial cells via the angiotensin-converting enzyme 2 (ACE2) pathway and aggravate endothelial dysfunction due to endothelitis, apoptosis, and lymphocytic and mononuclear infiltrating cells [41]. Endothelial cell injury and/or activation may lead to an imbalance of the coagulation system and thromboembolic complications associated with ischemic organ damage and consequently to high morbidity and mortality [42]. Increased ACE2 expression in bronchial epithelium and alveolar cells from diabetic patients increases SARS-CoV-2 infection [43]. ACE2 and transmembrane protease serine 2 (TMPRSS2) expression in islet cells may also promote SARS-CoV-2-mediated metabolic dysregulation due to cell death by necroptosis and immune cell infiltration [44] and reduced number of insulin-secretory granules in ß-cells and impaired glucose-stimulated insulin secretion [45], yet others did not find ACE2 expression in endocrine cells within the pancreas [46]. However, ACE2 expression in other tissues may contribute to insulin resistance, such as adipose tissue, where there is a positive correlation of ACE2 expression in subcutaneous and visceral fat and body mass index and, therefore, obesity [47], and in skeletal muscle cells, where ACE2 expression is associated with direct and indirect effects of SARS-CoV-2 [48].

Unexpectedly, diabetic individuals presented a lower frequency of anosmia (Supplementary Table S1). Surveillance analyses documented anosmia as a symptom not associated with the risk of hospitalization, indicating a lower severity of COVID-19 [49]. Although SARS-CoV-2 enters olfactory neuroepithelium via ACE2 receptor and TMPRSS2 [50] and causes anosmia, we can speculate that chronic hyperglycemia might have caused damage to nerve fibers and olfactory network and contributed ultimately to reducing the occurrence of this symptom. However, further studies are warranted to address anosmia frequency and evolution in diabetic individuals.

Our study has some limitations, including the number of patients, retrospective analyses, and the lack of other laboratory parameters that are correlated to COVID-19 outcomes, either at admission or longitudinally. In addition, our cohort has not received COVID-19 vaccination and the potential limitations (or not) of generalizability of the study findings to a vaccinated population warrant further investigation.

## Conclusion

Collectively, our data highlight the importance of early evaluation and identification of risk factors of COVID-19 progression and CFR for appropriately risk-stratifying kidney transplant recipients with DM, which may be extended to non-diabetics, during the pandemic. Encouraging healthy practices and strict glucose control in diabetic kidney transplant recipients and early identification of individuals at potential risk for COVID-19 progression and mortality are of paramount importance to mitigate adverse outcomes in this population during the pandemic.

## Data Availability

The original contributions presented in the study are included in the article/Supplementary Material, further inquiries can be directed to the corresponding author.

## Abbreviations

AKI, acute kidney injury; ALT, alanine aminotransferase; AST, aspartate aminotransferase; BMI, body mass index; CI, confidence interval; CFR, case fatality rate; CKD-EPI, chronic kidney disease epidemiology collaboration; COPD, chronic obstructive pulmonary disease; COVID-19, Coronavirus disease 2019; CRP, C-reactive protein; DM, diabetes mellitus; eGFR, estimated glomerular filtration rate; FBG, fasting blood glucose; HbA1c, glycated hemoglobin; HD, haemodialysis; ICU, intensive care unit; IMV, invasive mechanical ventilation; LDH, lactate dehydrogenase; O_2_, supplemental oxygen; OR, odds ratio; PSM, propensity score matching; ROC, receiver operating characteristic; SOT, solid-organ transplantation.
