# Supplementary material for: COVID-19 in Kidney Transplant Recipients With Diabetes Mellitus: A Propensity Score Matching Analysis
Source: Transpl Int. 2022 Jul 25;35:10375. doi: 10.3389/ti.2022.10375 (PMC9357874; doi:10.3389/ti.2022.10375)
Supplement: Supplementary file 1 [file DataSheet1.docx]

**Supplementary file.**

**Figure 1S.** Flowchart of patients included for selection of study patients.

**Supplementary file.**

**Figure 1S.** Flowchart of patients included for selection of study patients.


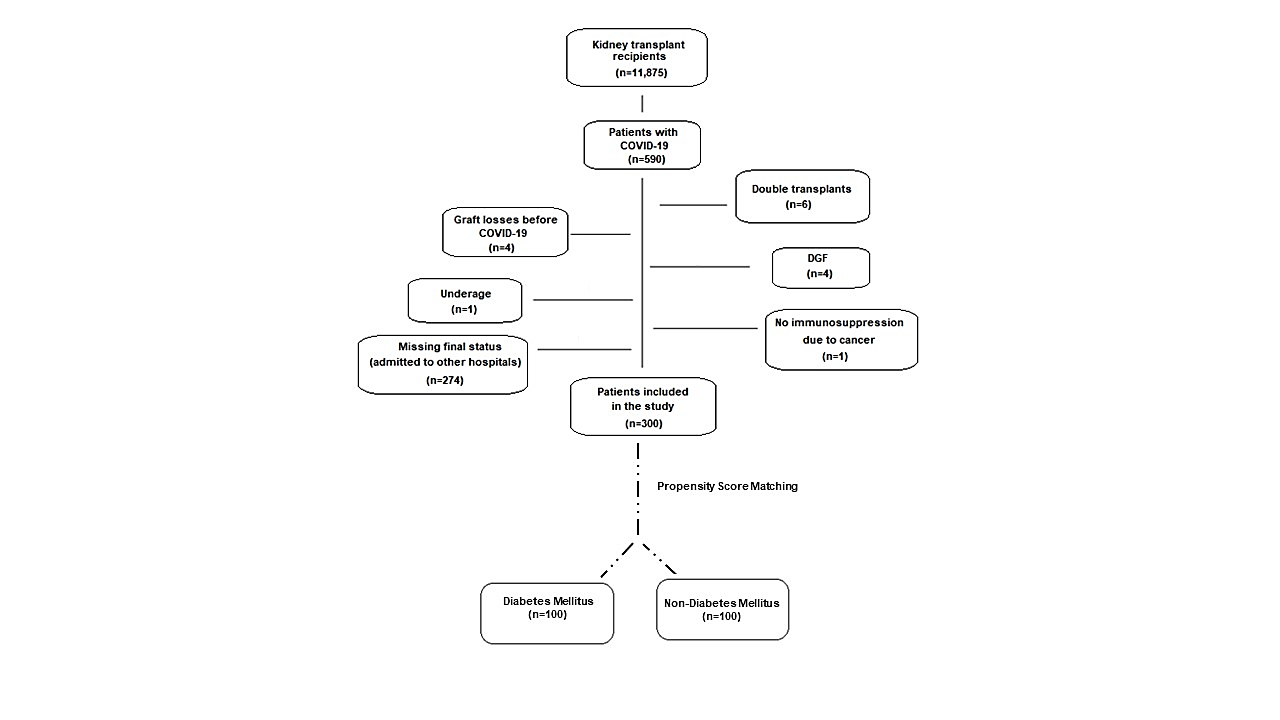


**Table 1S. Clinical and epidemiological characteristics and symptoms on admission in kidney transplant recipients with diabetes mellitus (DM) and without DM.**

| **Variables** | **DM (+)**  **(n=117, 39%)** | **DM (-)**  **(n=183, 61%)** | **TOTAL**  **(n=300, 100%)** | ***P*** | **OR** | **95% CI** | |
| --- | --- | --- | --- | --- | --- | --- | --- |
|  |  |  |  |  |  | **Lower** | **Upper** |
| Age (years) | 56.9±10.3 | 49.6±12.5 | 52.5±12.2 | ***0.0001*** | 1.055 | 1.032 | 1.078 |
| Male (n, %) | 64 (54.7) | 108 (59.0) | 172 (57.3) | 0.461 | 0.839 | 0.525 | 1.339 |
| Race (n, %)  White  Black/brown | 66 (56.4)  46 (39.3) | 118 (64.5)  64 (35.0) | 184 (61.3) 110 (36.7) | 0.162 | 0.713 | 0.444 | 1.146 |
| Obesity (n, %) | 31 (26.5) | 34 (18.6) | 65 (21.7) | 0.116 | 1.565 | 0.895 | 2.735 |
| BMI (kg/m^2^) | 27.4±5.1 | 26.6±4.7 | 26.9±4.9 | 0.198 | 1.033 | 0.983 | 1.084 |
| Hypertension (n, %) | 100 (85.5) | 124 (67.8) | 224 (74.7) | ***0.001*** | 2.799 | 1.535 | 5.102 |
| COPD (n, %) | 5 (4.3) | 4 (2.2) | 9 (3.0) | 0.310 | 1.998 | 0.525 | 7.598 |
| Smoking (n, %) | 28 (23.9) | 34 (18.6) | 62 (20.7) | 0.329 | 1.337 | 0.746 | 2.395 |
| Heart disease (n, %) | 21 (17.9) | 11 (6.0) | 32 (10.7) | ***0.002*** | 3.420 | 1.582 | 7.395 |
| Neoplasia (n, %) | 7 (6.0) | 14 (7.7) | 21 (7.0) | 0.582 | 0.768 | 0.301 | 1.964 |
| Liver disease (n, %) | 5 (4.3) | 4 (2.2) | 9 (3.0) | 0.310 | 1.998 | 0.525 | 7.598 |
| Autoimune disease (n, %) | 3 (2.6) | 5 (2.7) | 8 (2.7) | 0.930 | 0.937 | 0.220 | 3.996 |
| Transplant time (months) | 68.0 | 91.0 | 81.5 | 0.220 | 0.998 | 0.995 | 1.001 |
| Donor type (n, %)  Live  Deceased | 22 (18.8)  95 (81.2) | 63 (34.4)  120 (65.6) | 85 (28.3) 215 (71.7) | ***0.004*** | 2.267 | 1.301 | 3.949 |
| Fever (n, %) | 74 (63.2) | 107 (58.5) | 181 (60.3) | 0.410 | 1.222 | 0.759 | 1.970 |
| Chills (n, %) | 74 (63.2) | 107 (58.5) | 181 (60.3)) | 0.410 | 1.222 | 0.759 | 1.970 |
| Cough (n, %) | 70 (59.8) | 115 (62.8) | 185 (61.7) | 0.714 | 0.914 | 0.565 | 1.479 |
| Dyspnoea (n, %) | 64 (54.7) | 98 (53.6) | 162 (54.0) | 0.846 | 1.047 | 0.657 | 1.669 |
| Runny nose (n, %) | 23 (19.7) | 50 (27.3) | 73 (24.3) | 0.133 | 0.651 | 0.372 | 1.139 |
| Headache (n, %) | 19 (16.2) | 46 (25.1) | 65 (21.7) | 0.070 | 0.577 | 0.319 | 1.046 |
| Myalgia (n, %) | 49 (41.9) | 91 (49.7) | 140 (46.7) | 0.184 | 0.729 | 0.456 | 1.163 |
| Diarrhea (n, %) | 43 (36.8) | 65 (35.5) | 108 (36.0) | 0.828 | 1.055 | 0.651 | 1.709 |
| Anosmia (n, %) | 26 (22.2) | 63 (34.4) | 89 (29.7) | ***0.025*** | 0.544 | 0.320 | 0.926 |

BMI: body mass index; COPD: chronic obstructive pulmonary disease.

All values are mean and standard deviation, except the variable transplant time, which is median.**Table 2S. Laboratory data and outcomes in kidney transplant recipients with diabetes mellitus (DM) and without DM.**

| **Laboratory data** | **DM (+)**  **(n=117, 39%)** | | **DM (-)**  **(n=183, 61%)** | **TOTAL**  **(n=300, 100%)** | | ***P*** | **OR** | **95% CI** | | |
| --- | --- | --- | --- | --- | --- | --- | --- | --- | --- | --- |
|  |  |  |  |  |  |  |  | **Lower** | **Upper** | |
| Basal eGFR | | 48.3±24.4 | 49.4±24.2 | 49.0±24.2 | | 0.689 | 0.998 | 0.988 | 1.008 | |
| eGFR on admission | | 37.8±22.9 | 36.9±21.3 | 37.2±21.9 | | 0.728 | 1.002 | 0.991 | 1.013 | |
| Previous FBG (mg/dL) | | 159.6±88.3 | 93.9±18.9 | 119.1±65.0 | | ***0.0001*** | 1.038 | 1.027 | 1.049 | |
| Previous Hb1Ac (%) | | 8.1±2.2 | 5.7±0.7 | 6.9±2.0 | | ***0.0001*** | 5.246 | 3.316 | 8.298 | |
| CRP (mg/dL)* | | 7.8 | 4.7 | 5.7 | | ***0.007*** | 1.042 | 1.011 | 1.074 | |
| LDH (U/L)* | | 289.0 | 282.5 | 287.0 | | 0.726 | 1.000 | 0.999 | 1.002 | |
| Lymphocytes (mm³)* | | 728.0 | 740.5 | 738.0 | | 0.304 | 1.000 | 1.000 | 1.000 | |
| D-dimer (µg/L)* | | 1.2 | 1.2 | 1.2 | | 0.441 | 1.028 | 0.958 | 1.102 | |
| AST (U/L)* | | 27.0 | 30.0 | 28.0 | | 0.377 | 0.996 | 0.988 | 1.005 | |
| ALT (U/L)* | | 20.5 | 22.0 | 21.0 | | 0.468 | 0.996 | 0.985 | 1.007 | |
| **Outcomes** | |  |  |  | |  |  |  | | |
| Death (n, %) | | 46 (39.3) | 43 (23.5) | 89 (29.7) | | ***0.004*** | 2.109 | 1.274 | 3.493 | |
| ICU (n, %) | | 64 (54.7) | 76 (41.5) | 140 (46.7) | ***0.026*** | | 1.700 | 1.065 | | 2.714 |
| O_2_ (n, %) | | 72 (61.5) | 91 (49.7) | 163 (54.3) | ***0.046*** | | 1.618 | 1.009 | | 2.593 |
| IMV (n, %) | | 52 (44.4) | 50 (27.3) | 102 (34.0) | ***0.002*** | | 2.128 | 1.306 | | 3.469 |
| AKI (n, %) | | 74 (63.2) | 100 (54.6) | 174 (58.0) | 0.142 | | 1.428 | 0.888 | | 2.298 |
| Stage 1 | | 15 (12.8) | 23 (12.6) | 38 (12.7) | 0.949 | | 1.023 | 0.510 | | 2.052 |
| Stage 2 | | 4 (3.4) | 12 (6.6) | 16 (5.3) | 0.246 | | 0.504 | 0.159 | | 1.603 |
| Stage 3 | | 55 (47.0) | 65 (35.5) | 120 (40.0) | ***0.048*** | | 1.610 | 1.004 | | 2.584 |
| HD (n, %) | | 51 (43.6) | 58 (31.7) | 109 (36.3) | ***0.037*** | | 1.665 | 1.030 | | 2.692 |

eGFR: estimated glomerular filtration rate (in mL/min/1.73 m²); FBG: fasting blood glucose; Hb1Ac: glycated haemoglobin; CRP: C-reactive protein; LDH: lactate dehydrogenase; AST: aspartate aminotransferase; ALT: alanine aminotransferase; ICU: intensive care unit; O_2_: use of supplemental oxygen; IMV: invasive mechanical ventilation; AKI: acute kidney injury; HD: hemodialysis. All variables are means and standard deviations, except the variables CRP, LDH, Lymphocytes, D-dimer, AST and ALT, which are medians.

**Table 3S. Cox regression analysis and 60-days death pre-PSM (propensity score matching) in kidney transplant recipients with diabetes mellitus (DM) and without DM (n=300).**

| **Variables** | **HR** | ***P*** | **95% CI** | |
| --- | --- | --- | --- | --- |
|  |  |  | **Lower** | **Upper** |
| Age | 1.05 | ***<0.001*** | 1.03 | 1.07 |
| White ethnicity | 1.05 | 0.9 | 0.60 | 1.82 |
| Male | 0.94 | 0.8 | 0.55 | 1.61 |
| Body mass index | 1.01 | 0.8 | 0.95 | 1.07 |
| Live donor | 0.63 | 0.3 | 0.29 | 1.40 |
| Smoking | 1.06 | 0.9 | 0.58 | 1.93 |
| Hypertension | 1.57 | 0.2 | 0.79 | 3.15 |
| eGFR (basal) | 1.0 | 0.6 | 0.99 | 1.01 |
| eGFR: estimated glomerular filtration rate |  |  |  |  |
|  |  |  |  |  |
|  |  |  |  |  |

**Table 4S. Immunosuppression in kidney transplant recipients with diabetes mellitus (DM) and without DM after applying the propensity-score matching (PSM).**

| **Immunosuppression** | **DM (-)**  **(N=100)** | | **DM (+)**  **(N=100)** | ***P*** |
| --- | --- | --- | --- | --- |
|  |  |  |  |  |
| Immunosuppressive regimen | |  |  | ***0.001*** |
|  | |  |  |  |
| Tacrolimus + Azathioprine | | 13 (13%) | 22 (22%) |  |
| Tacrolimus + Mycophenolate  Tacrolimus + mTORi  Other | | 54 (54%)  2 (2%)  31 (31%) | 54 (54%)  11 (11%)  13 (13%) |  |
| Trough level (ng/mL) | |  |  |  |
|  | |  |  |  |
| Tacrolimus | | 7.4 (5.9, 10.2) | 7.9 (5.7, 10.2) | > 0.9 |
| Cyclosporine  Everolimus  Sirolimus | | 79 (61, 138)  4 (4.0, 4.0)  7.7 (6.2, 9.8) | 86 (39, 136)  7.0 (6.0, 8.65)  6.8 (5.9, 11.4) | 0.7  0.2  0.6 |
|  | |  |  |  |
|  | |  |  |  |
| Dose (mg/day)  Tacrolimus  Cyclosporine | | 6.0 (3.0, 8.0)  150 (100, 175) | 6.0 (3.0, 8.0)  125 (75, 175) | 0.7  0.8 |
| Everolimus  Sirolimus | | 3.0 (2.5-3.5)  3.0 (2.5, 3.25) | 3.0 (2.5-3.5)  2.0 (2.0-2.25) | > 0.9  0.4 |
| Azathioprine  Mycophenolate Sodium    Imunosupressive regimen modification  No modification  Complete suspension  Reduction of Azathioprine or Mycophenolate  Suspension of Azathioprine or Mycophenolate  Reduction of calcineurin inhibitor  Suspension of calcineurin inhibitor | | 125 (100, 150)  720 (720, 1440)  38 (38%)  47 (47%)  0 (0%)  16 (16%)  0 (0%)  1 (1%) | 100 (100, 100)  720 (720, 1440)  32 (32%)  47 (47%)  0 (0%)  22 (22%)  0 (0%)  0 (0%) | 0.4  > 0.9  0.4  > 0.9  0.3  > 0.9 |
|  | |  |  |  |

mTORi: mammalian target of rapamycin inhibitor
